# Highly Pathogenic Avian Influenza A(H5N1) Clade 2.3.4.4b Virus in Poultry, Benin, 2021

**DOI:** 10.3201/eid2812.221020

**Published:** 2022-12

**Authors:** Idrissa Nonmon Sanogo, Fidelia Djegui, Yao Akpo, Corneille Gnanvi, Gabriel Dupré, Adam Rubrum, Trushar Jeevan, Pamela McKenzie, Richard J. Webby, Mariette F. Ducatez

**Affiliations:** Université de Ségou, Ségou, Mali (I.N. Sanogo);; Institut national de recherche pour l’agriculture, l’alimentation et l’environnement, Ecole Nationale Vétérinaire de Toulouse, Toulouse, France (I.N. Sanogo, G. Dupré, M.F. Ducatez);; Laboratoire de Diagnostic vétérinaire et de Sérosurveillance, Parakou, Benin (F. Djegui);; Direction de l’Elevage, Cotonou, Benin (Y. Akpo, C. Gnanvi);; St. Jude Children’s Research Hospital, Memphis, Tennessee, USA (A. Rubrum, T. Jeevan, P. McKenzie, R.J. Webby)

**Keywords:** highly pathogenic avian influenza, H5N1 subtype, influenza, respiratory infections, zoonoses, viruses, genetic characterization, phylogenetic analysis, poultry, Benin, antigenic cartography, One Health

## Abstract

In August 2021, we detected highly pathogenic avian influenza A(H5N1) clade 2.3.4.4b viruses in poultry in southern Benin. The isolates were genetically similar to H5N1 viruses of clade 2.3.4.4b isolated during the same period in Africa and Europe. We also found evidence for 2 separate introductions of these viruses into Benin.

Highly pathogenic avian influenza (HPAI) viruses represent a major threat to animal and public health. HPAI A/Goose/Guangdong/1/96-lineage subtype H5N1 viruses first emerged in southern China in 1996, and their descendants have since evolved into different phylogenetic clades causing large outbreaks in poultry and wild birds worldwide (*1*). Since first being detected in Nigeria in 2006, HPAI H5N1 viruses have been responsible for numerous outbreaks in many countries in Africa, causing high mortality in domestic and wild birds over the past 15 years (*2*,*3*). 

Beginning in January 2021, outbreaks caused by HPAI H5N1 clade 2.3.4.4b virus have been reported in many countries in West Africa, including Mali, Nigeria, Niger, and Senegal (*3*). Clade 2.3.4.4 H5 viruses are of particular concern because of their potential for reassortment and ability to cross the species barrier and infect new hosts, including humans, seals, and foxes (*4*,*5*). In addition, increasing infectivity of these HPAI H5 viruses in humans could accelerate their adaptation to human-to-human transmission, which might increase the possibility for emergence of a novel influenza strain with pandemic potential (*6*). In August 2021, HPAI H5N1 viruses were detected in poultry in the southern region of Benin. In this study, we carried out genetic and antigenic analyses to investigate the origin of the virus and its relationship with viruses detected in neighboring countries.

## The Study

During August–September 2021, high mortality was reported in chickens from poultry farms in Seme-Podji and Ouidah Provinces in southern Benin. Clinical signs in the affected birds included prostration, respiratory distress, severe diarrhea, depression, and lack of coordination. Based on the epidemiologic status of avian influenza in the region, which involved outbreaks in neighboring countries Nigeria and Niger, and the possibility of infected birds being moved across country borders through legal or illegal trade of poultry, HPAI was suspected. 

We collected 468 samples (organ tissues and oropharyngeal swabs) from among poultry at 6 infected farms and poultry markets. We performed one-step real-time reverse transcription PCR (rRT-PCR) targeting the influenza A virus H5 and N1 genes and detected avian influenza H5N1 virus RNA in 11 samples. We isolated viruses from positive samples in embryonated eggs, then performed molecular characterization by whole-genome sequencing using an Illumina MiSeq system (https://www.illumina.com) as described elsewhere (*7*). We obtained 5 H5N1 virus isolates from which we generated full-genome sequences and designated them A/poultry/Benin/21-A-08-009-O/2021, A/poultry/Benin/21-A-09-031-O/2021, A/poultry/Benin/21-A-09-033-O/2021, A/poultry/Benin/21-A-09-034-O/2021, and A/poultry/Benin/21-A-09-035-O/2021; we deposited all sequences into GenBank (accession nos. ON870413–47). The isolates shared a high nucleotide sequence identity of 98.62%–99.91% and amino acid similarity among one another. 

To determine the origin of the Benin H5N1 viruses, we performed phylogenetic analysis for all 8 genomic segments from the 5 isolates using the maximum-likelihood method and inferred phylogenetic trees using IQ-TREE version 2.1.2 software (http://www.iqtree.org) with automatic model selection and 1,000 nonparametric bootstrap replicates (*8*). Our analysis revealed that the Benin HPAI H5N1 viruses were closely related to H5N1 viruses isolated in Nigeria in 2021 and, to a lesser extent, to viruses detected in Lesotho in 2021 and Europe in 2020–2021, suggesting a possible transmission route from Nigeria to Benin. The topology of the hemagglutinin (HA) gene tree (Appendix Figure 1) indicated that the H5N1 viruses from Benin belonged to clade 2.3.4.4b (*9*). Phylogenetic trees based on the other gene segments (Appendix Figures 2–8) indicated that no reassortment event had occurred within the Benin H5N1 isolates. However, A/poultry/Benin/21-A-09-031-O/2021 did not cluster with the other HPAI H5N1 from Benin, suggesting 2 independent introductions of the virus into the country. 

**Figure 1:**
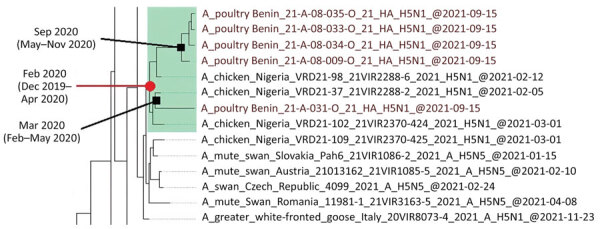
Maximum clade credibility tree of the hemagglutinin (HA) gene of highly pathogenic avian influenza A(H5N1) viruses from Benin (red) and reference viruses. Green box highlights viruses sharing the same common ancestor with the Benin isolates. The time to the most common ancestor and the 95% highest posterior density intervals are indicated for the relevant nodes.

To estimate the time to the most recent common ancestor (tMRCA), we conducted Bayesian Markov Chain Monte Carlo sampling in BEAST version 1.10.4 (https://beast.community) (*10*) under the general time-reversible plus invariant sites plus Γ4 nucleotide substitution model, using an uncorrelated log-normal relaxed clock and Bayesian Skygrid coalescent prior as described elsewhere (*11*). The H5N1 isolates from Benin originated from the same common ancestor as the H5N1 viruses isolated in Nigeria in 2021 (Figure 1); based on the HA maximum clade credibility tree, the average tMRCA of these viruses was estimated as February 2020 (95% highest posterior density interval December 2019–April 2020). The likelihood of 2 independent introductions of the H5N1 viruses into Benin was supported by the tMRCAs estimated for each gene segment (Appendix Table 1), the first involving A/poultry/Benin/21-A-09-031-O/2021, during March–April 2020, and the second involving the other 4 Benin isolates, during July–August 2020. 

**Figure 2:**
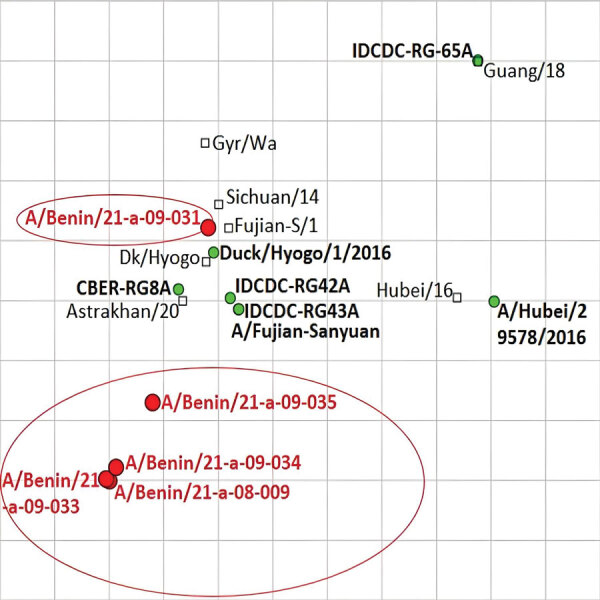
Antigenic map of highly pathogenic avian influenza A(H5N1) viruses from Benin based on hemagglutination inhibition data (Appendix Table). Circles indicate viruses and squares antiserum; red indicates viruses characterized in this study, and green dots indicate reference viruses. The spacing between grid lines is 1 unit of antigenic distance, corresponding to a 2-fold dilution of antiserum in the hemagglutination inhibition assay.

To study the antigenic profiles of the HPAI H5N1 isolated in Benin, we performed a hemagglutination inhibition (HI) assay against reference ferret postinfection serum (Appendix Table 2), as described elsewhere (*12*). We used HI results to build an antigenic map (Figure 2) using ACMACS software (https://acmacs-web.antigenic-cartography.org). The antigenic map revealed 2 slightly distinct antigenic profiles for Benin H5N1 viruses. A/poultry/Benin/21-A-09-031-O/2021 was antigenically similar to most of the World Health Organization candidate vaccine viruses used in the HI assay, especially to A/Sichuan/26221/2014-like (IDCDC-RG42A) and A/duck/Hyogo/1/2016 (NIID-001). In contrast, the other 4 Benin isolates that clustered together in the phylogenetic trees were 2–3 log_2_ away from the candidate viruses, suggesting a good but lower level of cross-reactivity. The most antigenically similar candidate virus to these 4 Benin isolates was A/Astrakhan/3212/2020-like (CBER-RG8A). Amino acid sequence analysis showed that the H5N1 viruses from Benin had multiple basic amino acids motif (PLREKRRKR/GLF) at the HA cleavage site, characteristic of HPAI viruses. In addition, we detected a mammalian adaptation mutation in the polymerase basic 1 segment (113V) of the 5 Benin isolates, suggesting potential for these viruses to replicate more efficiently in mammalian cells (*13*). 

## Conclusions 

Although several outbreaks occurred in the region, relatively little information was available on the genetic and antigenic diversity of HPAI H5N1 clade 2.3.4.4b viruses in poultry in West Africa. We characterized 5 HPAI H5N1 clade 2.3.4.4b viruses from Benin highly similar to viruses detected earlier in neighboring Nigeria. It is therefore likely that the Benin H5N1 viruses originated from the region because of the movement of infected poultry and poultry products across neighboring countries (*14*). The initial introduction of H5N1 viruses into the wider region might be related to the arrival of migratory birds. In fact, the tMRCA of Benin H5N1 viruses in 2021 corresponded to the period (January–March) when Eurasian migratory birds are present in West Africa (*15*). Furthermore, the relationship between Benin H5N1 isolates and viruses detected earlier in Europe corroborates this hypothesis. 

HPAI H5N1 clade 2.3.4.4b viruses represent a major concern for West Africa and have demonstrated the potential for spreading more widely in the region. Therefore, to monitor virus evolution and promptly identify viruses with increased zoonotic potential, the countries of West Africa require regional collaboration for long-term surveillance and whole-genome sequencing of HPAI viruses in both humans and animals. 

Appendix Additional information about highly pathogenic avian influenza A(H5N1) virus in poultry, Benin.
