# Supplementary material for: Highly Pathogenic Avian Influenza A(H5N1) Clade 2.3.4.4b Virus in Poultry, Benin, 2021
Source: Emerg Infect Dis. 2022 Dec;28(12):2534–7. doi: 10.3201/eid2812.221020 (PMC9707597; doi:10.3201/eid2812.221020)
Supplement: Appendix — Additional information about highly pathogenic avian influenza A(H5N1) virus in poultry, Benin. [file 22-1020-Techapp-s1.pdf]

Article DOI: https://doi.org/10.3201/eid2812.221020

Highly Pathogenic Avian Influenza A(H5N1) Clade 2.3.4.4b Virus in Poultry, Benin, 2021

Appendix

Appendix Table 1. tMRCA of each gene segment of the 2021 Benin H5N1 viruses*

Segment	A/poultry/Benin/21-A-09-031-O/2021, tMRCA (95% HPD range)†	A/poultry/Benin/21-A-08-009-O/2021, A/poultry/Benin/21-A-09-033-O/2021, A/poultry/Benin/21-A-09-034-O/2021, A/poultry/Benin/21-A-09-035-O/2021, tMRCA (95% HPD range)†
PB2	Apr (Mar–May)	Aug (Aug–Oct)
PB1	Mar (Jan–Jul)	Jul (Mar–Sep)
PA	Apr (Feb–May)	Aug (Jun–Oct)
HA	Mar (Feb–May)	Sep (May–Oct)
NP	Mar (Feb–May)	Jul (Mar–Aug)
NA	Apr (Feb–May)	Jul (Apr–Sep)
M	Aug (May–Oct)	Aug (May–Oct)
NS	Apr (Feb–May)	Aug (May–Oct)

*PB2, Polymerase basic protein 2; PB1, polymerase basic protein 1; PA, polymerase acidic protein; HA, hemagglutinin; NP, nucleoprotein; NA, neuraminidase; M, matrix protein; NS, nonstructural protein; tMRCA, time to most recent common ancestor; HPD, highest posterior density.
†All dates 2020

Appendix Table 2. Hemagglutination inhibition assay of clade 2.3.4.4 viruses (with 0.5% turkey red blood cells)

Virus	Subtype	Clade	Sichuan /14	Fujian-S /1	Astrakhan /20*	Gyr/Wa	Hubei /16	Dk/Hyogo	Guang /18
Reference antigen									
IDCDC-RG42A (A/Sichuan/26221/2014-like)	H5N6	2.3.4.4a	320	160	160	40	10	640	<10
A/Fujian-Sanyuan/21099/2017 (CNIC-21099)	H5N6	2.3.4.4b	160	160	160	80	0	320	<10
CBER-RG8A (A/Astrakhan/3212/2020-like)	H5N6	2.3.4.4b	160	160	320	160	<10	320	<10
IDCDC-RG43A (A/gyrfalcon/WA/41088-6/2014-like)	H5N6	2.3.4.4c	160	160	160	80	<10	320	<10
A/Hubei/29578/2016 (CNIC-29578)	H5N6	2.3.4.4d	<10	<10	<10	<10	160	10	10
A/duck/Hyogo/1/2016 (NIID-001)	H5N6	2.3.4.4e	320	320	320	320	10	640	<10
IDCDC-RG-65A (A/Guangdong/18SF020/2018-like)	H5N6	2.3.4.4f	<10	<10	<10	<10	10	10	160
Test antigen									
A/poultry/Benin/21-A-08-009-O/2021	H5N1	2.3.4.4b	<10	10	40†	10	<10	40	<10
A/poultry/Benin/21-A-09-031-O/2021	H5N1	2.3.4.4b	640	320	320‡	640	<10	640	<10
A/poultry/Benin/21-A-09-033-O/2021	H5N1	2.3.4.4b	<10	10	40†	10	<10	40	<10
A/poultry/Benin/21-A-09-034-O/2021	H5N1	2.3.4.4b	<10	10	80§	10	<10	40	<10
A/poultry/Benin/21-A-09-035-O/2021	H5N1	2.3.4.4b	<10	20	160‡	20	<10	160	<10

*Closest candidate viruses
†8 fold
‡≤2 fold
§4 fold

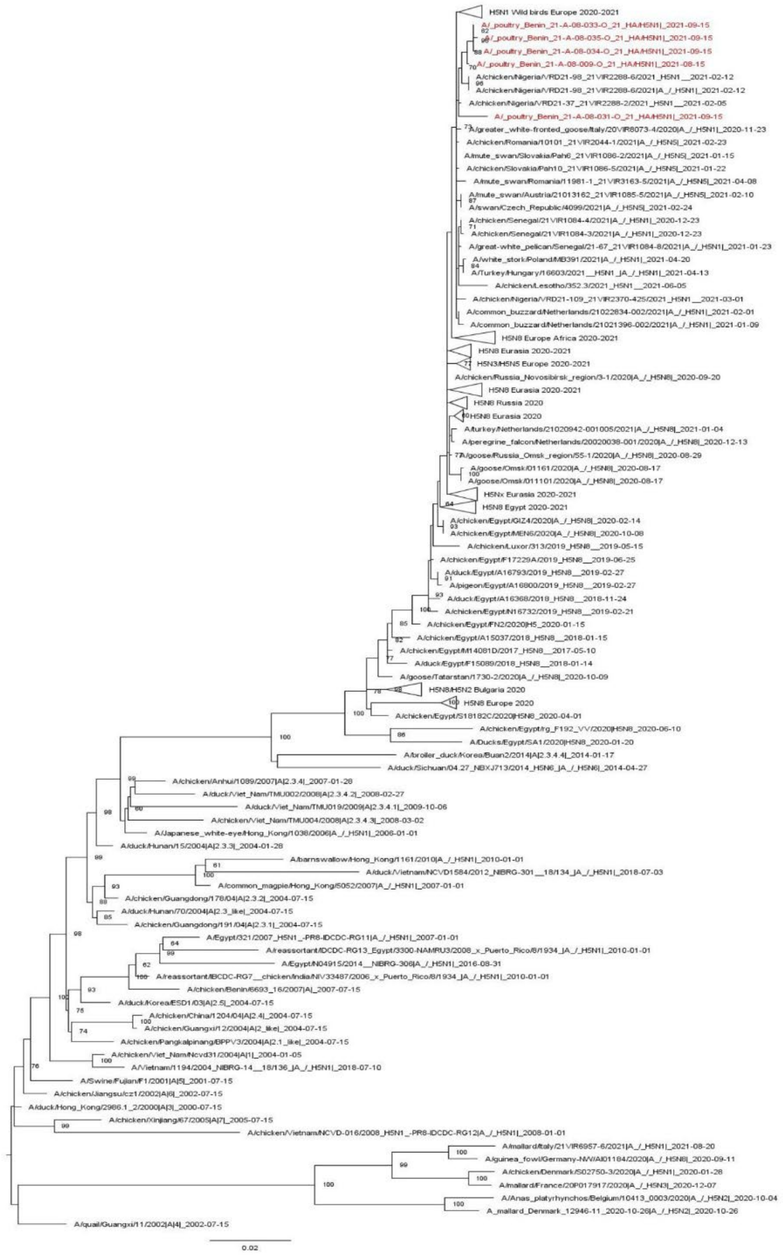

Appendix Figure 1. Maximum likelihood phylogenetic tree of the hemagglutinin (HA) gene. H5N1 virus sequences from Benin are indicated in red. Bootstrap values ≥ 60% are shown near the nodes. Scale bar indicates the average number of nucleotide substitutions per site.

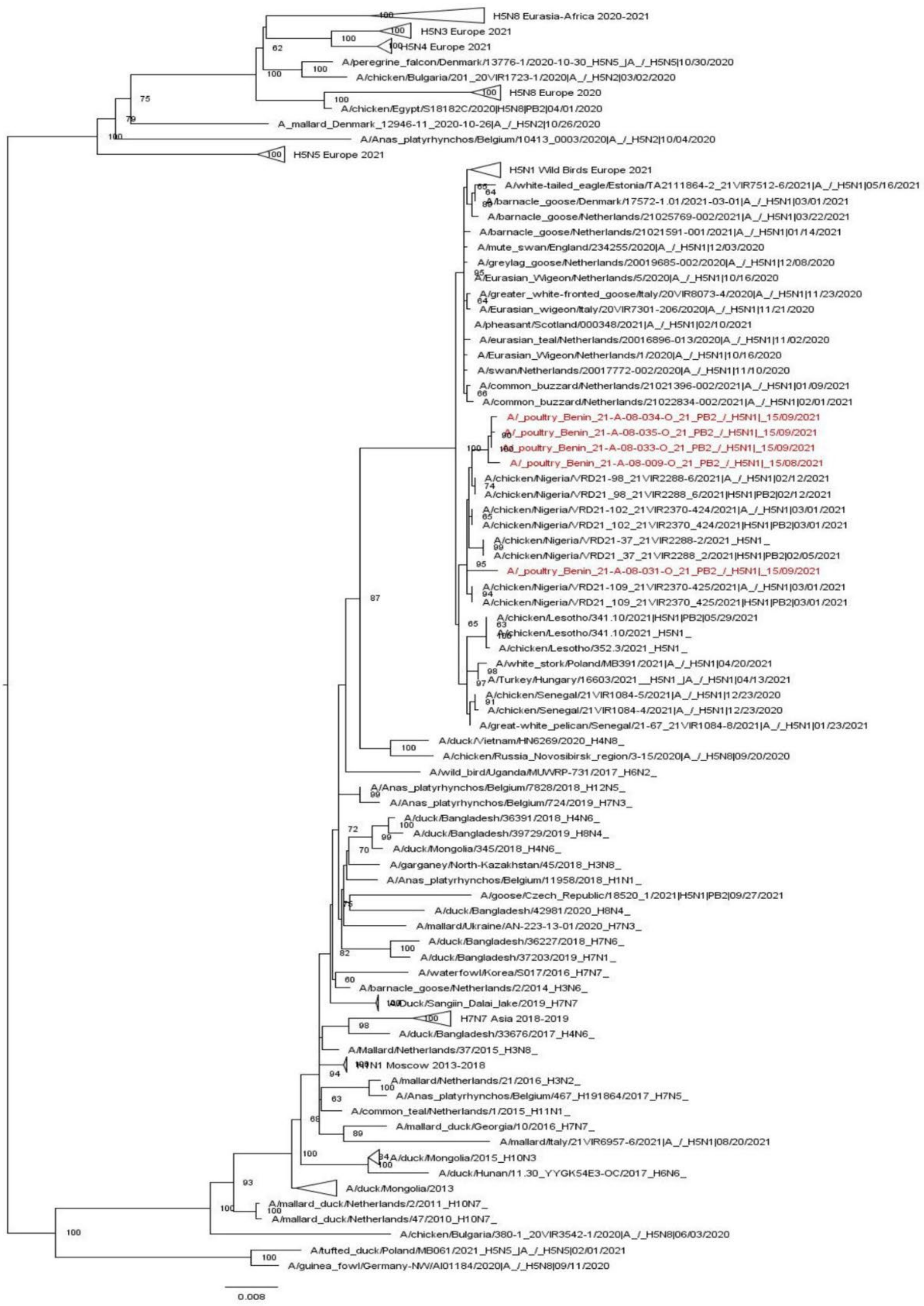

Appendix Figure 2. Maximum Likelihood phylogenetic tree of the polymerase basic 2 (PB2) gene. H5N1 virus sequences from Benin are indicated in red. Bootstrap values ≥ 60% are shown near the nodes. Scale bar indicates the average number of nucleotide substitutions per site.

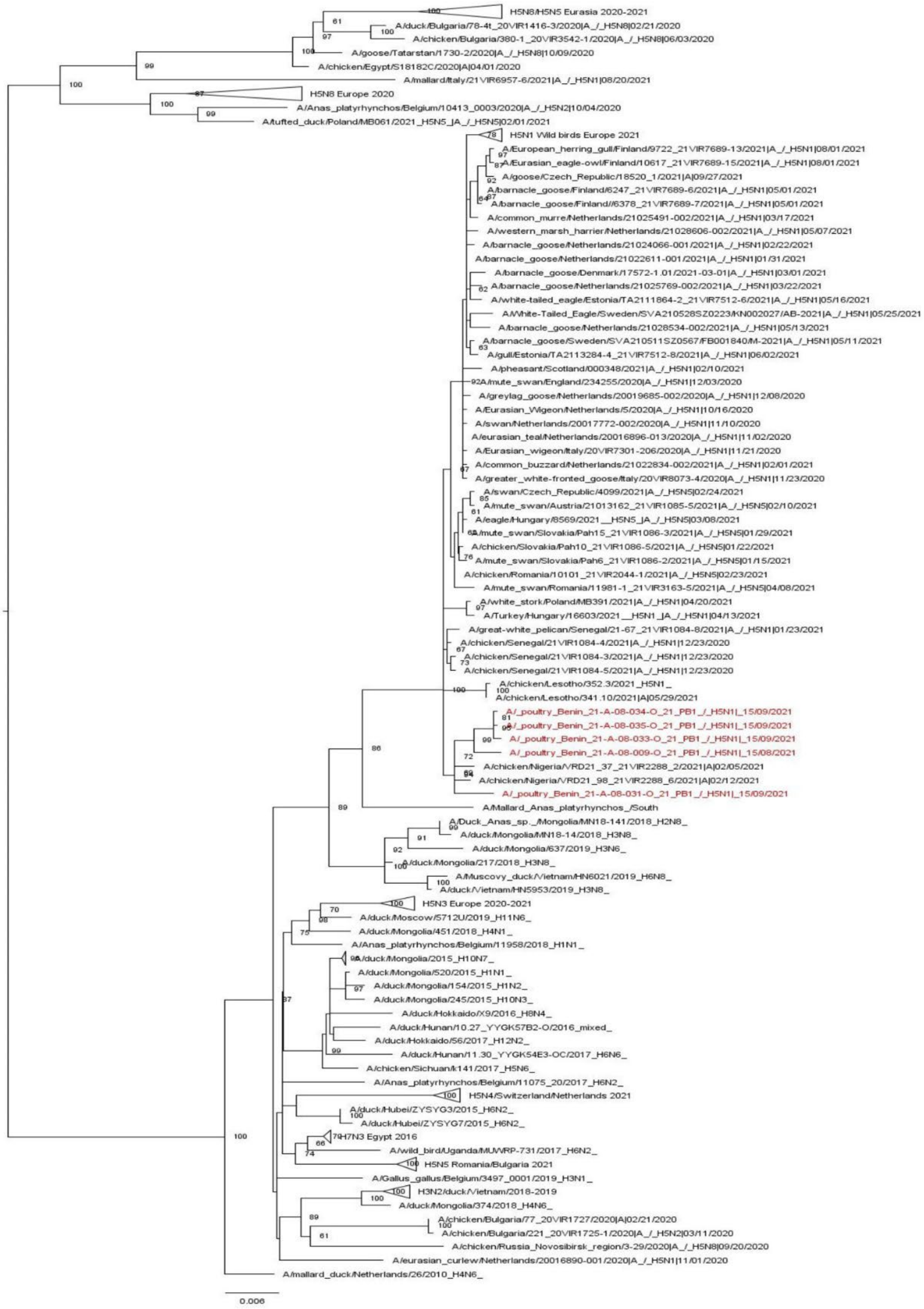

Appendix Figure 3. Maximum likelihood phylogenetic tree of the polymerase basic 1 (PB1) gene. H5N1 virus sequences from Benin are indicated in red. Bootstrap values ≥ 60% are shown near the nodes. Scale bar indicates the average number of nucleotide substitutions per site.

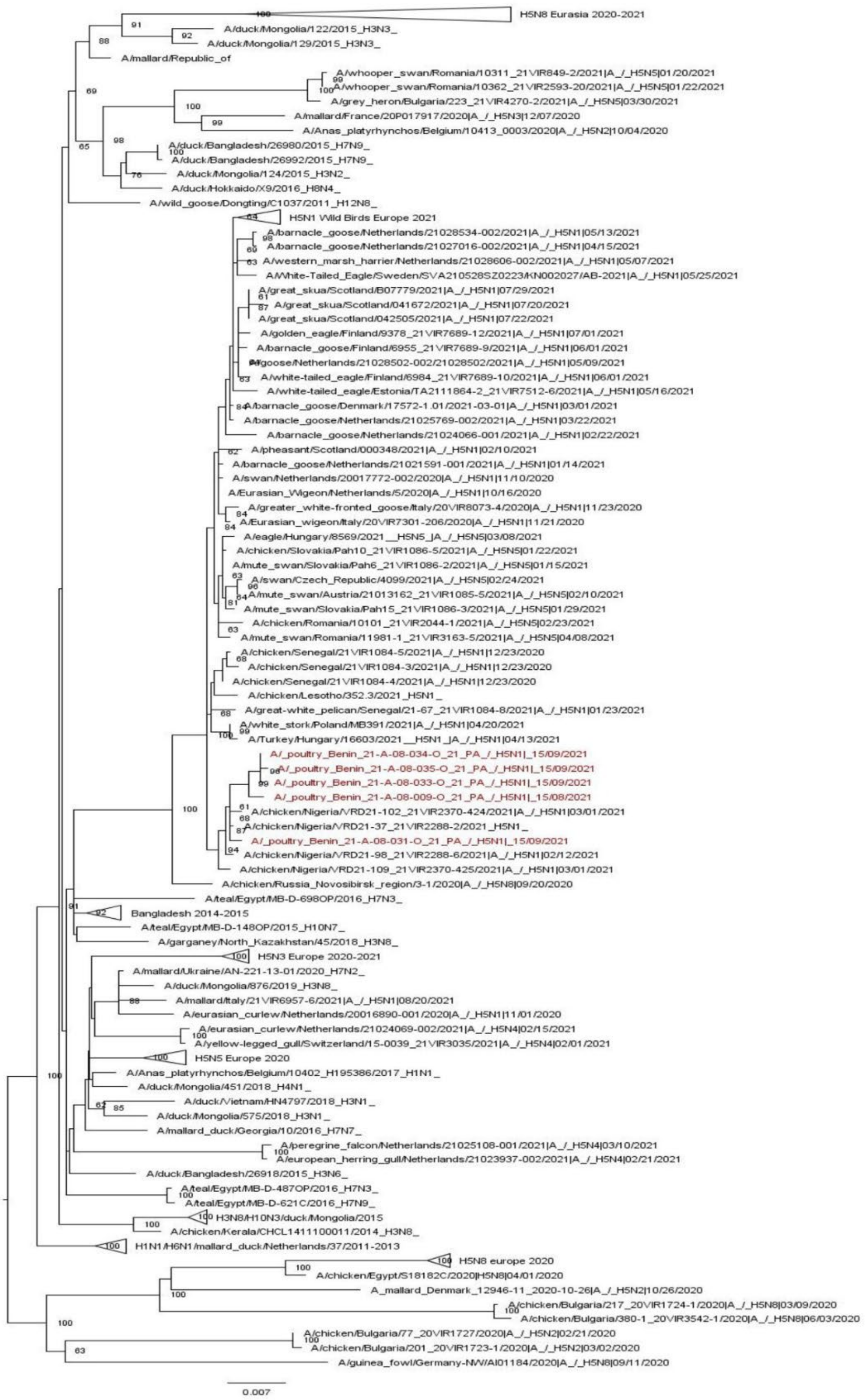

Appendix Figure 4. Maximum likelihood phylogenetic tree of the polymerase acidic (PA) gene. H5N1 virus sequences from Benin are indicated in red. Bootstrap values ≥ 60% are shown near the nodes. Scale bar indicates the average number of nucleotide substitutions per site.

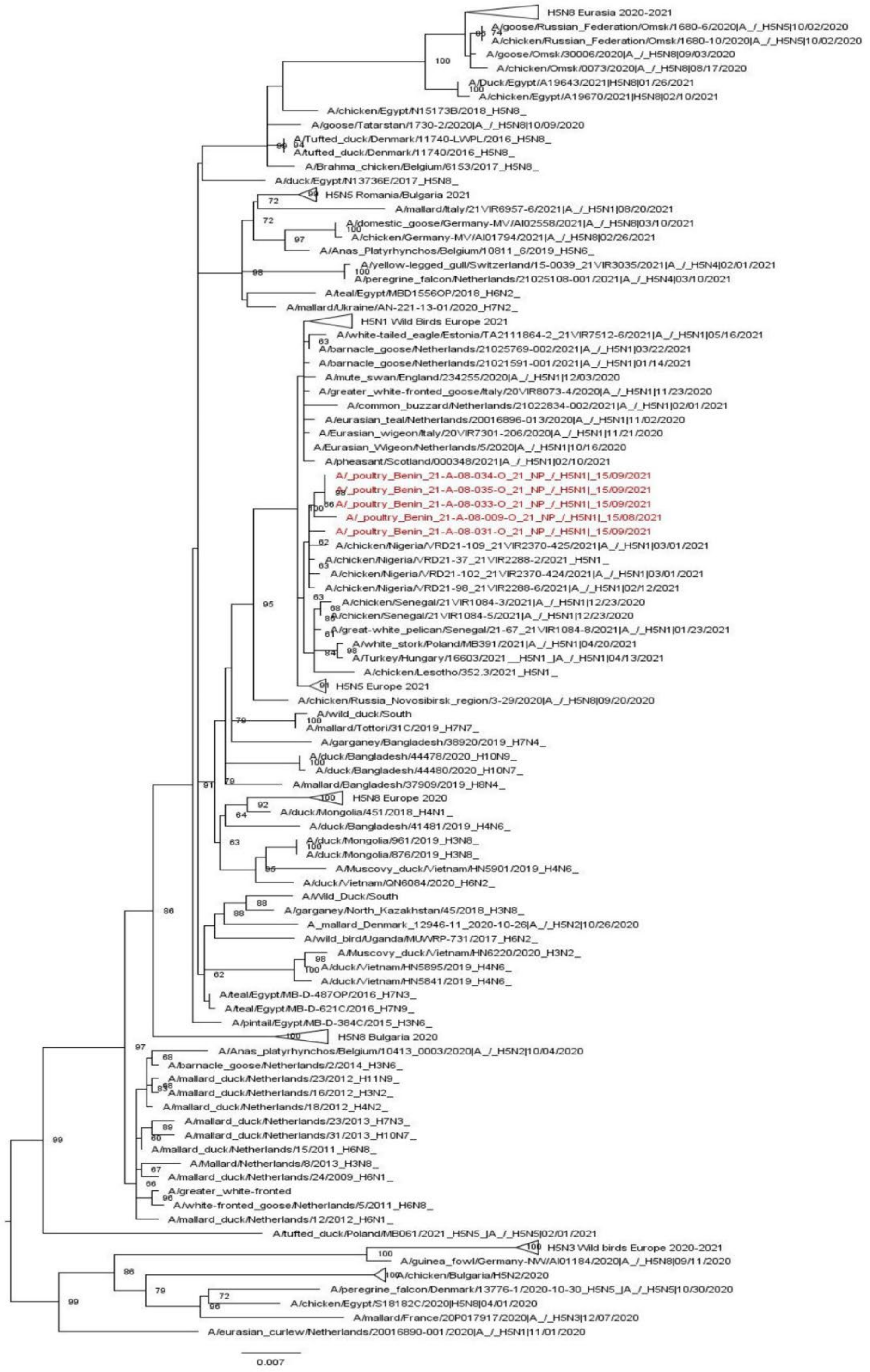

Appendix Figure 5. Maximum likelihood phylogenetic tree of the nucleoprotein (NP) gene. H5N1 virus sequences from Benin are indicated in red. Bootstrap values ≥ 60% are shown near the nodes. Scale bar indicates the average number of nucleotide substitutions per site.

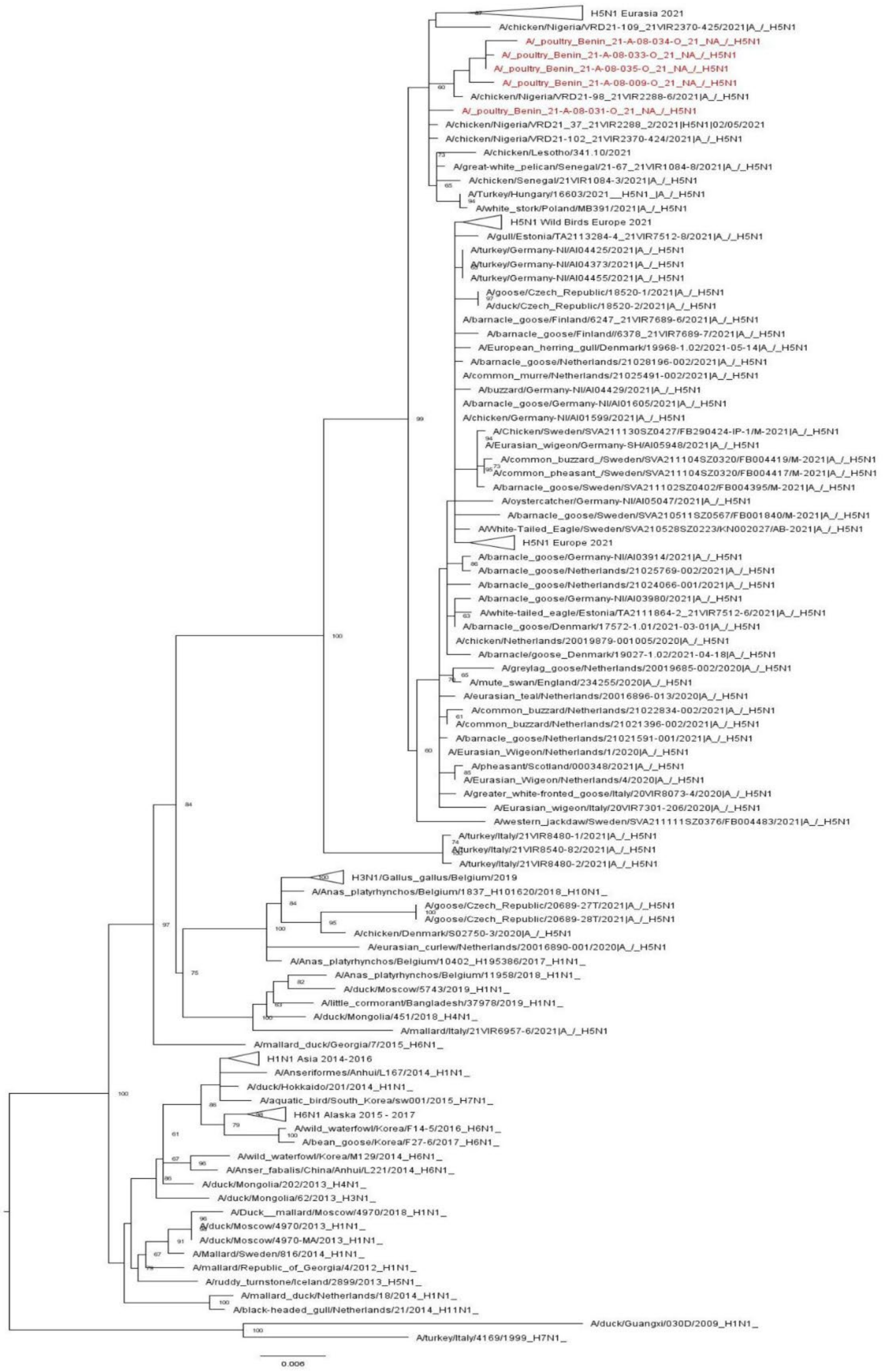

Appendix Figure 6. Maximum likelihood phylogenetic tree of the neuraminidase (NA) gene. H5N1 virus sequences from Benin are indicated in red. Bootstrap values ≥ 60% are shown near the nodes. Scale bar indicates the average number of nucleotide substitutions per site.

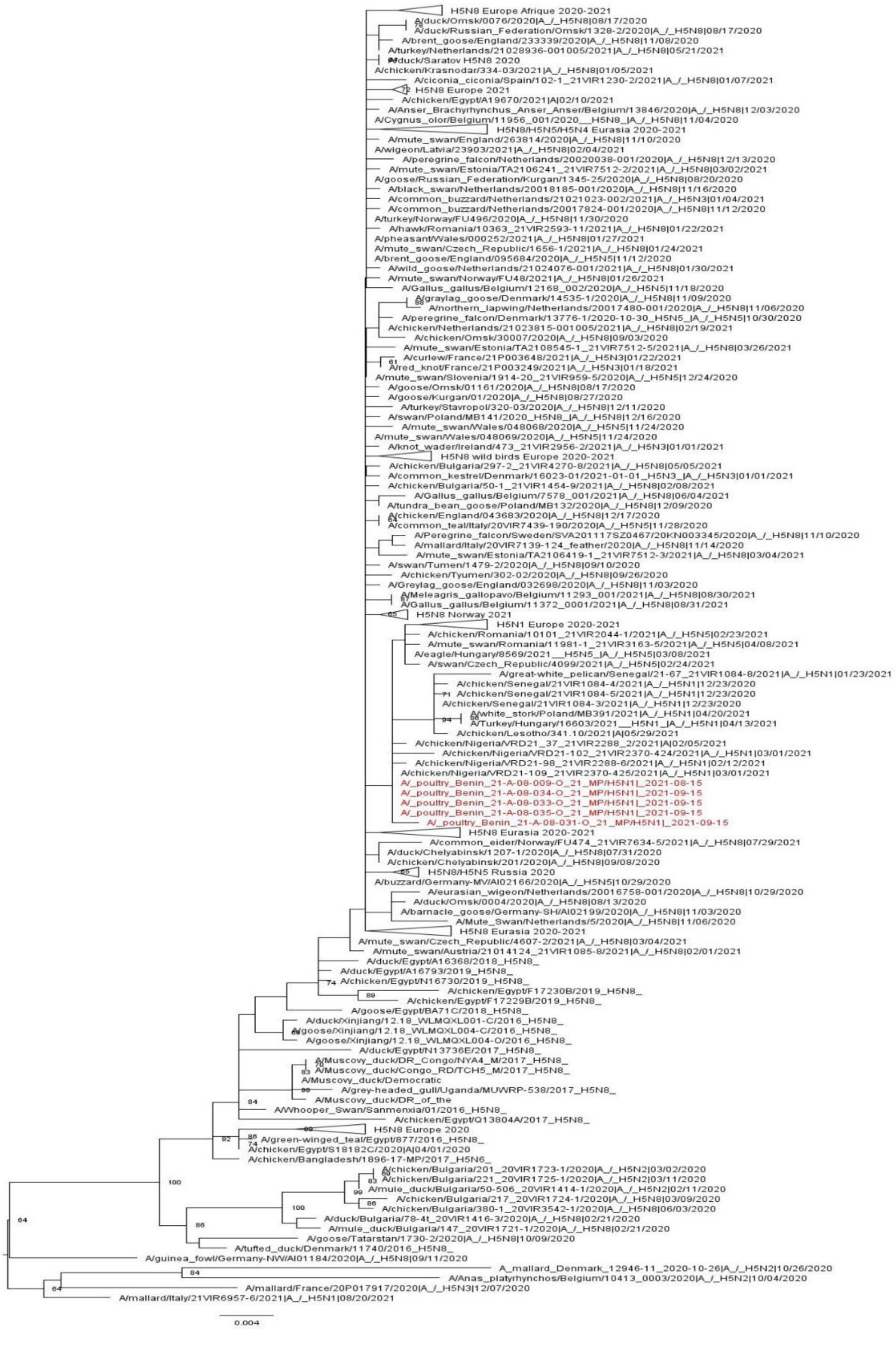

Appendix Figure 7. Maximum likelihood phylogenetic tree of the matrix protein (M) gene. H5N1 virus sequences from Benin are indicated in red. Bootstrap values ≥ 60% are shown near the nodes. Scale bar indicates the average number of nucleotide substitutions per site.

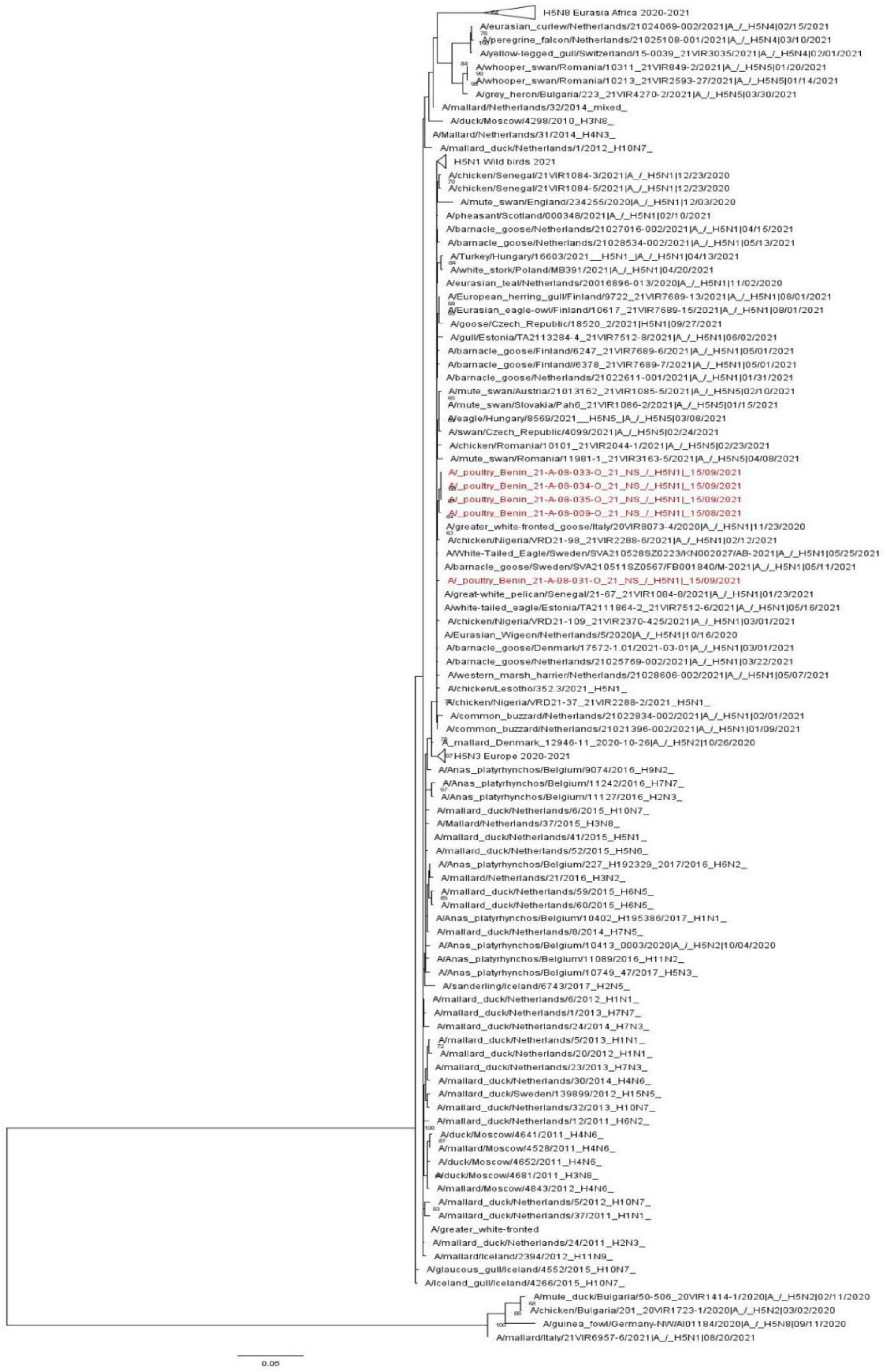

Appendix Figure 8. Maximum Likelihood phylogenetic tree of the Nonstructural protein (NS) gene. H5N1 virus sequences from Benin are indicated in red. Bootstrap values ≥ 60% are shown near the nodes. Scale bar indicates the average number of nucleotide substitutions per site.
